# Facial Identification at a Virtual Reality Airport

**DOI:** 10.1177/2041669519863077

**Published:** 2019-07-12

**Authors:** Hannah M. Tummon, John Allen, Markus Bindemann

**Affiliations:** School of Psychology, University of Kent, Canterbury, UK

**Keywords:** face, person, identification, matching, virtual reality, passport, airport

## Abstract

Person identification at airports requires the comparison of a passport photograph with its bearer. In psychology, this process is typically studied with static pairs of face photographs that require identity-match (same person shown) versus mismatch (two different people) decisions, but this approach provides a limited proxy for studying how environment and social interaction factors affect this task. In this study, we explore the feasibility of virtual reality (VR) as a solution to this problem, by examining the identity matching of avatars in a VR airport. We show that facial photographs of real people can be rendered into VR avatars in a manner that preserves image and identity information (Experiments 1 to 3). We then show that identity matching of avatar pairs reflects similar cognitive processes to the matching of face photographs (Experiments 4 and 5). This pattern holds when avatar matching is assessed in a VR airport (Experiments 6 and 7). These findings demonstrate the feasibility of VR as a new method for investigating face matching in complex environments.

## Introduction

Passport officers at airports and national borders are widely required to verify the identity of travellers by comparing their faces to passport photographs. People seeking to avoid detection at such security controls may attempt to do so by acting as impostors, using valid identity documents that belong to other persons who are of sufficiently similar facial appearance. In psychology, this task has been studied extensively as unfamiliar face matching (for reviews, see Fysh & Bindemann, 2017a; Jenkins & Burton, 2008, 2011; Robertson, Middleton, & Burton, 2015). In experiments in this field, observers are typically required to match pairs of face photographs, which are presented in isolation on blank backgrounds, and have to decide whether these depict the same person or two different people.

This general approach has been successful for isolating and understanding a range of important factors, such as *observer* characteristics. For example, pairwise face-matching experiments have been used to assess individual differences in performance (e.g., Bindemann, Avetisyan, & Rakow, 2012; Bobak, Dowsett, & Bate, 2016; Bobak, Hancock, & Bate, 2016; Megreya & Burton, 2006a), to compare untrained observers with passport officers (White, Kemp, Jenkins, Matheson, & Burton, 2014; Wirth & Carbon, 2017) and different groups of professionals, such as facial review staff and facial examiners (White, Dunn, Schmid, & Kemp, 2015; see also Phillips et al., 2018; White, Phillips, Hahn, Hill, & O’Toole, 2015), and to assess observers familiar and unfamiliar with the target identities (Bruce, Henderson, Newman, & Burton, 2001; Ritchie et al., 2015), as well as those with impairments in face matching (White, Rivolta, Burton, Al-Janabi, & Palermo, 2017). Similarly, such controlled laboratory experiments have been employed to study how the characteristics of *stimuli* affect face matching, by exploring factors such as image quality (e.g., Bindemann, Attard, Leach, & Johnston, 2013; Strathie & McNeill, 2016), the addition of paraphernalia and disguise (Henderson, Bruce, & Burton, 2001; Kramer & Ritchie, 2016; Wirth & Carbon, 2017), and variation in viewpoint (Estudillo & Bindemann, 2014), camera distance (Noyes & Jenkins, 2017), and facial appearance across photographs (e.g., Bindemann & Sandford, 2011; Megreya, Sandford, & Burton, 2013).

While this research has advanced understanding of face matching considerably, these paradigms provide a limited proxy for studying how the environment and social interaction might affect this task. In real-life environments, passport officers may, for example, resort to nonfacial cues, such as body language, to support identification decisions (Rice, Phillips, Natu, An, & O’Toole, 2013; Rice, Phillips, & O’Toole, 2013). Similarly, environmental factors, such as the presence of passenger queues, might impair identification by exerting time pressure on passport officers (see, e.g., Bindemann, Fysh, Cross, & Watts, 2016; Fysh & Bindemann, 2017b; Wirth & Carbon, 2017) or competition for attention (see, e.g., Bindemann, Burton, & Jenkins, 2005; Bindemann, Sandford, Gillatt, Avetisyan, & Megreya, 2012; Megreya & Burton, 2006b). The impact of such factors is likely to be huge but not captured by current laboratory paradigms and practically impossible to study in real life owing to the importance of person identification at passport control.

As a compromise, a few studies have moved beyond highly controlled laboratory paradigms to study this task in simplified field settings (e.g., Kemp, Towell, & Pike, 1997; Megreya & Burton, 2008; White et al., 2014). White et al. (2014), for example, examined passport officers' matching accuracy under live conditions, in which target identities were presented in person and compared with a face photograph on a computer screen. Such paradigms are valuable for assessing whether limitations in face-matching accuracy are also observed in interpersonal interaction but are logistically challenging. Moreover, such setups do not adequately capture the complexity of real-life passport control environments and cannot provide the control that experimenters might desire to manipulate environment and social interaction factors accurately for psychological experimentation.

In this project, we propose a potential solution to these problems, by examining face matching in virtual reality (VR). In recent years, this technology has developed rapidly to provide affordable high-capability VR equipment. With VR, viewers can be immersed in detailed, interactive, and highly controllable three-dimensional (3D) environments that conventional laboratory experiments cannot provide. However, this approach is completely new to face matching. In this article, we therefore report an exploratory series of experiments to investigate the potential of VR for increasing our understanding of face matching. Our overall aim is to provide a foundation for further face-matching research with VR, by demonstrating that this approach can capture the face processes that are currently studied with more simplistic laboratory approaches.

In VR, people are represented by animated 3D avatars, on which we superimpose the two-dimensional (2D) faces of real persons. In the first phase of experimentation, we assess the quality of the resulting person avatars in a tightly controlled laboratory task, in which these 3D avatars are presented back as isolated 2D images, to establish that these capture the identities from which they were derived (Experiments 1 to 3). In the second phase of the study, we compare identity matching of these avatars with two established laboratory tests of face matching (Experiments 4 and 5). In the final phase, identification of avatars is then assessed in an immersive 3D VR airport environment (Experiments 6 and 7).

## Phase 1: Avatar Face Construction and Validation

We begin with a description of the construction of the person avatars for our experimentation. The initial stimulus sets consisted of 129 male and 88 female professional German sportspeople. As these identities were required to be unfamiliar to our participants, a pretest was carried out to ensure these people were not generally recognisable to U.K. residents. A list of the identities was presented to 20 students who were asked to cross the names of anyone who they would recognise. Identities familiar to two or more people were excluded. From those who remained, 50 male and 50 female identities were selected for avatar creation. We employed two full-face portrait photographs for each of these sportsmen and women, which were obtained via Google searches.

The person avatars for this study were created by combining these face photographs with an existing database of person avatars (see www.kent.ac.uk/psychology/downloads/avatars.pdf) with graphics software (Artweaver Free 5). The internal features of the face were cut as a selection from the photograph and overlaid onto the base avatar’s graphics file. The size of the selection was altered to best map the features onto the positions of the base avatar’s features. This was then smoothed around the edges and skin colour adjusted to blend in with the base avatar. Note that the 3D structure of the avatar faces could not be adapted to that of the face photographs, as extraction of such shape information is limited from 2D images. This may be suboptimal for modelling face recognition, to which both texture and shape information contribute (e.g., O’Toole, Vetter, & Blanz, 1999). However, face recognition is also tolerant to dramatic manipulations of shape (see Bindemann, Burton, Leuthold, & Schweinberger, 2008; Hole, George, Eaves, & Rasek, 2002), and texture appears to be more diagnostic for face identification and face matching (see, e.g., Calder, Burton, Miller, Young, & Akamatsu, 2001; Hancock, Burton, & Bruce, 1996; Itz, Golle, Luttmann, Schweinberger, & Kaufmann, 2017). Therefore, our method for combining the 2D photographs with animated 3D avatars captures the most diagnostic information for identification. In addition, to mitigate for the fact that we could not incorporate original shape information, the same base avatar was employed for both face photographs of each identity. However, avatar elements such as clothing were changed to create two unique appearances for each instance of a person. Therefore, for each of the 100 identities retained, two avatars were created. For the experiments reported here, this pool of avatars provided sufficient stimuli to create identity-match pairs consisting of two avatars of the same person and identity mismatch pairs consisting of two avatars from different people.

**Figure 1.**
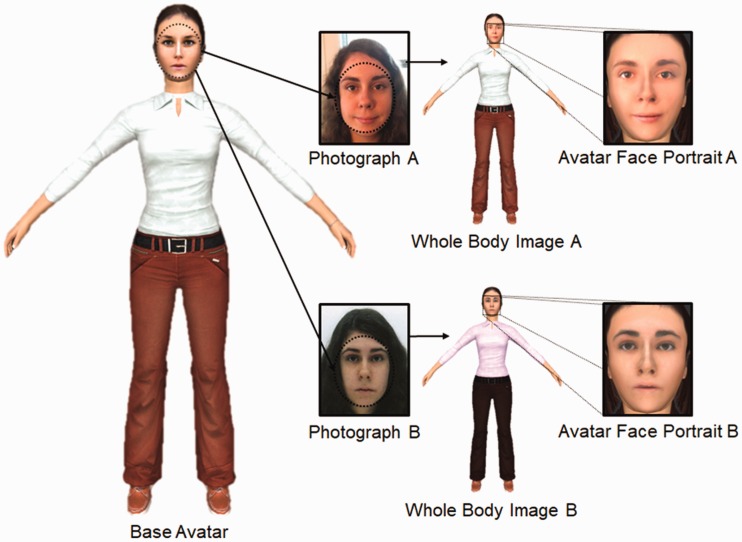
An illustration of avatar construction. 2D face photographs were superimposed on animated 3D avatar bodies, whose clothing could be adapted for different identities. 2D face portraits and full-body images were then derived from the 3D avatars for initial experimentation.

As an initial step, we sought to confirm that the resulting avatars adequately capture the identities of the face set. For this purpose, we recorded a 2D face portrait of each finished identity avatar. These images were constrained to reveal the internal facial features only (i.e., not hairstyle) and sized to 438 (w) × 563 (h) pixels at a resolution of 150 ppi. In addition, a 2D full-body image, which showed a frontal view of the avatar with arms outstretched, was also recorded and sized to 751 (w) × 809 (h) pixels at a resolution of 150 ppi. The procedure for avatar construction is illustrated in Figure 1.

## Experiment 1

The aim of Experiment 1 was to assess whether the production process of the avatar faces sufficiently captures the images and identities on which these are based. If so, then observers should be able to match these identities in a pairwise comparison. This was assessed with a face photograph-to-avatar matching test with three conditions. These comprised trials on which an avatar face portrait was paired with the original source face photograph (same-image identity match), trials on which an avatar face portrait was paired with a different face photograph of the same person (different-image identity match), and trials on which the avatar face portrait was paired with a face photograph of a different person (identity mismatch). Participants were asked to match these stimulus pairs according to whether they depicted the same person or two different people.

### Method

#### Participants

Thirty Caucasian participants (12 men, 18 women) with a mean age of 21.6 years (*SD* = 3.7 years), who reported normal or corrected-to-normal vision, were recruited at the University of Kent for course credit or a small payment. This sample size is directly comparable to face-matching studies using a broad range of paradigms (e.g., Bindemann et al., 2013; Megreya & Burton, 2007; White et al., 2017).

#### Stimuli and Procedure

Each participant was presented with 80 trials across 2 blocks, with each block comprising the following image-type trials. First, 10 same-image identity-match pairs were produced, which consisted of a 2D avatar face portrait and the high-quality face photograph used to create that avatar. Second, 10 different-image identity-match trials were included, in which the 2D avatar face portrait was combined with a different photograph of the same person. These trials did not consist of any of the identities shown in the same-image identity-match trials. Finally, 20 mismatch trials were created. In these, the 2D avatar face portrait was paired with a photograph of a different person, which was chosen by the experimenter (H. M. T.) for its general visual similarity.

The stimuli of the second block consisted of the same identity pairings as the first block (i.e., 10 same-image identity matches, 10 different-image identity matches, 20 mismatches) but with the reverse image-type pairings, as demonstrated in Figure 1. For example, if an observer saw Avatar Face Portrait A paired with Photograph B for an identity in Block 1, then in Block 2 for the same identity, Avatar Face Portrait B was paired with Photograph A. Thus, all participants saw each identity twice during the course of the experiment but each image (avatar face portrait or face photograph) only once. All of these images were presented on a white background, with the avatar face portrait to the left and the face photograph to the right of centre. Both images were sized to 70 mm (w) × 90 mm (h) and were presented 50 mm apart.

In the experiment, each trial began with a 1-second fixation cross, followed by a stimulus pair, which remained on screen until a matching decision had been made. Participants were asked to decide as accurately as possible whether a stimulus pair depicted the same person or two different people, by pressing one of two corresponding buttons on a standard computer keyboard. The experiment was presented using PsychoPy (Peirce, 2007), and stimulus identities were rotated around the conditions across observers. Block order was counterbalanced.

### Results

**Figure 2.**
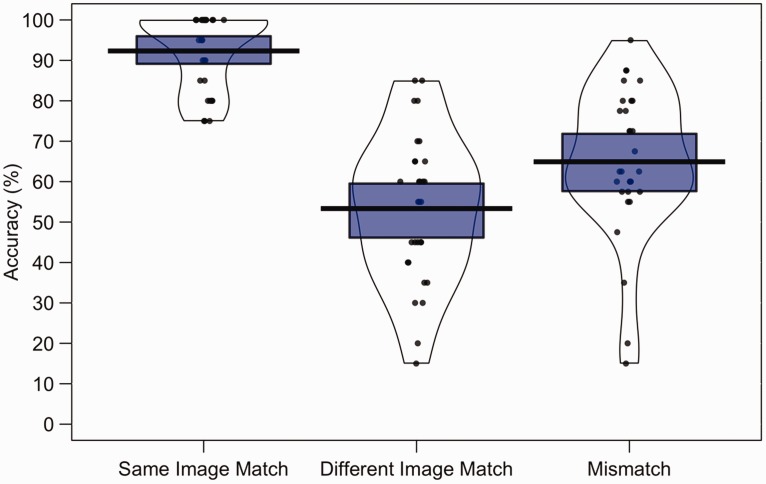
Percentage accuracy data for Experiment 1. The mean performance of each trial type is denoted by the black lines with the coloured boxes representing 95% confidence intervals. The black dots represent the accuracy of individual participants. The width of each violin represents the expected probability density of performance.

To assess performance, the percentage of accurate responses was calculated for all conditions. This is shown in Figure 2, which also illustrates individual performance. A one-factor analysis of variance (ANOVA) of these data showed an effect of trial type, *F*(2,58) = 37.83, *p* < .001, η_p_^2^ = .57, with paired-samples *t* tests (with alpha corrected to .017 [.05/3] for three comparisons) indicating higher accuracy on same-image identity-match trials (*M* = 92.3%, *SD* = 9.4) than different-image identity-match trials (*M* = 53.3%, *SD* = 18.3) and mismatch trials (*M* = 64.9%, *SD* = 18.7), *t*(29) = 13.73, *p* < .001, *d* = 2.65 and *t*(29) = 6.58, *p* < .001, *d* = 1.83, respectively. The difference in accuracy between different-image identity-match trials and mismatch trials was not reliable, *t*(29) = 1.87, *p* = .07, *d* = 0.62.

Considering the low accuracy for different-image identity-match trials and mismatch trials, a series of one-sample *t* tests was also conducted to determine whether accuracy was above chance (i.e., 50%) for the conditions. This was the case for same-image identity matches, *t*(29) = 24.79, *p* < .001, *d* = 6.32, and identity mismatches, *t*(29) = 4.38, *p* < .001, *d* = 1.12, but not for different-image identity matches, *t*(29) = 1.00, *p* = .33, *d* = 0.25. The data sets for all experiments reported here are available online as supplemental material.

### Discussion

This experiment shows that matching of avatar faces to their source face photographs is highly accurate, which indicates that image-specific identity information from these source images is captured well. By contrast, matching of avatar faces to a different photograph of the same person was difficult and did not reliably exceed the chance benchmark of 50%. Accuracy was also fairly low for identity mismatches, comprising pairings of avatar faces with face photographs of a different person. The low accuracy in these conditions is potentially problematic for adopting VR to study unfamiliar face matching, but it is possible that this is caused by the inclusion of same-image identity matches. While this condition was included here to assess the production process of the stimuli, it is typically not included in face-matching experiments (see, e.g., Fysh & Bindemann, 2018). Considering that these same-image stimulus pairs inevitably display much greater similarity than different-image identity matches and mismatches, the inclusion of this condition may have served to attenuate the perceived differences between these critical identity conditions, resulting in a reduction in accuracy. To address this possibility, only different-image identity matches and mismatches were employed in Experiment 2.

## Experiment 2

This experiment further assesses whether the production process of the avatars captures the identities on which these are based. In contrast to Experiment 1, this was assessed with only two conditions, comprising different-image identity matches and identity mismatches, to minimise the influence that same-image identity matches might exert on the classification of these conditions.

### Method

#### Participants

Thirty Caucasian participants from the University of Kent (10 men, 20 women), with a mean age of 19.6 years (*SD* = 1.5 years), participated in exchange for a small fee or course credit. None of these participants had participated in Experiment 1.

#### Stimuli and Procedure

Stimuli and procedure were identical to Experiment 1, except that same-image identity matches were excluded. All observers completed 2 blocks of 40 trials, comprising 20 different-image identity matches and 20 mismatches pairs in each block. As was the case in Experiment 1, Block 2 consisted of the reverse image-type stimulus pairings for the identities in Block 1. Once again, all trials began with a 1-second fixation cross and were presented in a randomised order, block order was counterbalanced, and accuracy of response was emphasised.

### Results

**Figure 3.**
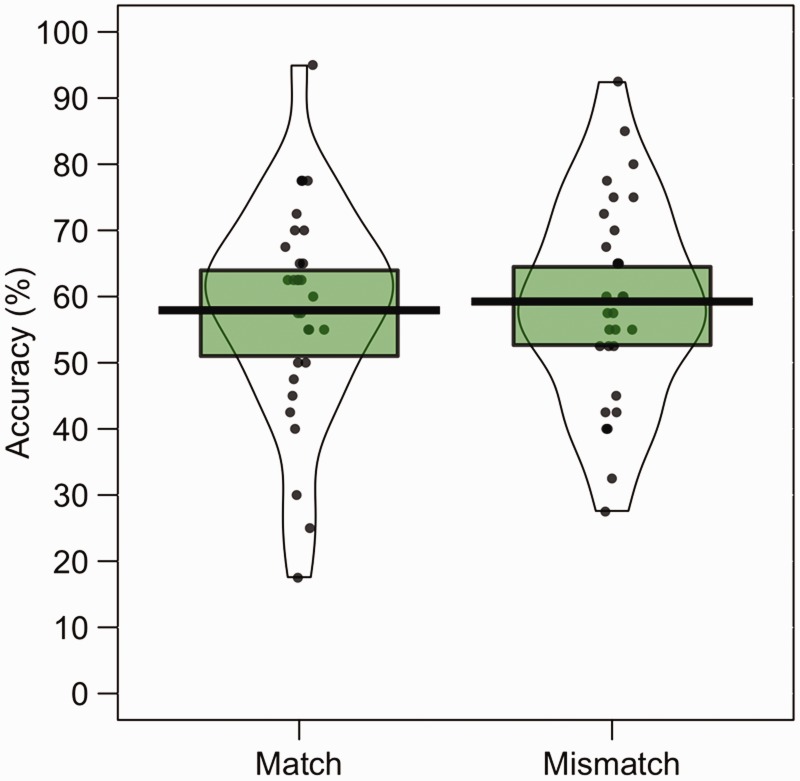
Percentage accuracy data for Experiment 2. The mean performance of each trial type is denoted by the black lines with the coloured boxes representing 95% confidence intervals. The black dots represent the accuracy of individual participants. The width of each violin represents the expected probability density of performance.

The percentage accuracy data for Experiment 2 are illustrated in Figure 3. A paired-sample *t* test of these data showed that accuracy was comparable for different-image identity-match trials (*M* = 57.9%, *SD =* 16.4) and mismatch trials (*M* = 59.3%, *SD =* 15.4), *t*(29) = 0.25, *p* = .80, *d* = 0.08. In addition, one-sample *t* tests revealed that performance in both conditions was above the chance level of 50%, with *t*(29) = 2.64, *p* = .01, *d* = 0.67 and *t*(29) = 3.28, *p* = .003, *d* = 0.84 for match and mismatch trials, respectively.

### Discussion

Experiment 1 revealed that the avatars capture the face source photographs sufficiently for accuracy on same-image identity-match trials to be high. Experiment 2 complements these findings by showing that accuracy for different-image identity matches and mismatches exceeds chance when these same-image trials are excluded. Different-image identity matches are a fundamental requirement for studying the identification of unfamiliar faces, to ensure that this task is not solved by using simple image-matching strategies (see, e.g., Burton, 2013; Jenkins & Burton, 2011). The data from Experiment 2 therefore provide initial evidence that avatar stimuli have the potential to provide a suitable substrate to study face identification processes in VR.

## Experiment 3

The two preceding experiments in this initial avatar validation phase have compared avatar face portraits with source photographs. These demonstrate that such avatar portraits capture the facial characteristics of their respective source photographs and can also be matched to a different photograph from which they were created to an above chance level. This final validation experiment separates these two image types to investigate whether performance of avatar-to-avatar facial comparisons is consistent with performance of photograph-to-photograph comparisons.

### Method

#### Participants

Thirty Caucasian participants from the University of Kent (1 man, 29 women), with a mean age of 19.2 years (*SD* = 2.0 years), participated in exchange for course credit. None of these participants had participated in any of the preceding experiments.

#### Stimuli and Procedure

The stimuli for this experiment consisted of the same 20 match and 20 mismatch identity pairings of Experiment 2, presented in 2 blocks (80 trials in total). However, rather than combining an avatar face portrait with a source photograph, Avatar Face Portraits A and B were paired together in one block of trials, while source Photographs A and B were paired together in a second block. As with the previous experiments, all trials began with a 1-second fixation cross and were presented in a randomised order. Block order was counterbalanced across participants, and accuracy of response was emphasised.

### Results

**Figure 4.**
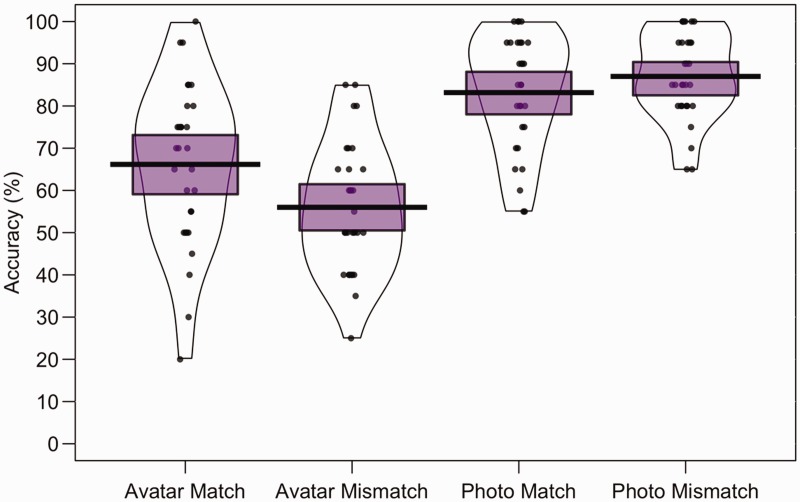
Percentage accuracy data for Experiment 3. The mean performance of each trial type is denoted by the black lines with the coloured boxes representing 95% confidence intervals. The black dots represent the accuracy of individual participants. The width of each violin represents the expected probability density of performance.

To compare performance across image type, the mean percentage accuracy of correct match and mismatch responses was calculated for all conditions. These data are illustrated in Figure 4. For avatar-to-avatar comparisons, accuracy was higher for match trials (*M* = 66.2%, *SD* = 19.1) than mismatch trials (*M* = 56.0%, *SD* = 15.4). The opposite pattern was observed for photograph-to-photograph comparison trials, with higher accuracy for mismatch trials (*M* = 87.0%, *SD* = 10.3) than for match trials (*M* = 83.2%, *SD* = 13.7). A 2 (image type: source photograph, avatar) × 2 (trial type: match, mismatch) within-subjects ANOVA of these data did not show a main effect of trial type, *F*(1, 29) = 0.55, *p* = .47, η_p_^2^ = .02, but revealed a main effect of image type, *F*(1, 29) = 219.55, *p* < .001, η_p_^2^ = .88, and an interaction between factors, *F*(1, 29) = 13.67, *p* < .001, η_p_^2^ = .32. A simple main effect of image type was found for match, *F*(1, 29) = 54.31, *p* < .001, η_p_^2^ = .65, and mismatch trials, *F*(1, 29) = 135.51, *p* < .001, η_p_^2^ = .82, due to higher accuracy for photograph than avatar matching. No simple main effects of trial type were found within avatar matching, *F*(1, 29) = 3.29, *p* = .08, η_p_^2^ = .10, and photograph matching, *F*(1, 29) = 1.17, *p* = .29, η_p_^2^ = .04.

One-sample *t* tests showed that match and mismatch accuracy for photographs exceeded chance (50%), *t*(29) = 13.22, *p* < .001, *d* = 3.37, and *t*(29) = 19.67, *p* < .001, *d* = 5.01, respectively. Importantly, this was also the case for match and mismatch trials with avatar portraits, *t*(29) = 4.62, *p* < .001, *d* = 1.18 and *t*(29) = 2.13, *p* = .04, *d* = 0.54.

Finally, accuracy for source photographs and avatar faces correlated on both match trials, *r* = .752, *p* < .001, and mismatch trials, *r* = .415, *p* < .05, indicating that matching of both stimulus types reflects the same underlying cognitive processes.

### Discussion

In contrast to Experiments 1 and 2, which examined photograph-to-avatar matching, the current validation experiment demonstrates that avatar faces also can be successfully matched to each other. Avatar matching was more difficult than matching pairs of face photographs, but this is unsurprising considering that the photographs reflect the original identity images. In addition, identities for mismatches were paired up based on avatar similarity, which should increase the difficulty of this task relative to matching of photographs also. Despite this, performance for avatar-to-avatar and photograph-to-photograph matching correlated well, indicating that both reflect the same underlying processes. The next phase of this study will explore this further, by comparing avatar matching with two established tests of face matching.

## Phase 2: Matching Avatars Versus Matching Face Photographs

The experiments of Phase 1 demonstrate that avatar identification is a difficult task and also indicate that avatar matching reflects similar processes to matching of face photographs. To examine this further prior to implementation in a VR environment, we sought to correlate matching of avatar face pairs with two tests of unfamiliar face matching in Phase 2, comprising the widely used Glasgow Face Matching Test (GFMT; Burton, White, & McNeill, 2010) and the newer Kent Face Matching Test (KFMT; Fysh & Bindemann, 2018). Of these tests, the GFMT represents a best case scenario to assess face-matching accuracy, by providing highly controlled, same-day photographic pairs of faces. The KFMT, on the other hand, provides a more challenging matching test, in which face pairs consist of a controlled face portrait and an uncontrolled image. Despite these differences, performance on the GFMT and KFMT correlates well. Here, we investigate whether such correlations exist also between these tests and the matching of avatar face pairs.

## Experiment 4

This experiment compared performance on the GFMT and KFMT, which required matching of photographs of faces, with the matching of pairs of avatar faces. Overall, performance should be best with the optimised stimuli of the GFMT than the more challenging KFMT. In addition, accuracy for the KFMT should be similar to avatar-to-avatar face matching, considering that both tests are based on different-day face images. The main aim here, however, was to correlate performance on these tasks to explore whether these capture the same identification processes.

### Method

#### Participants

The participants consisted of 30 Caucasian individuals (8 men, 22 women), with a mean age of 21.2 years (*SD* = 3.3 years), who were paid a small fee or given course credit. None of these participants had participated in the preceding experiments.

#### Stimuli and Procedure

#### *The GFMT*

**Figure 5.**
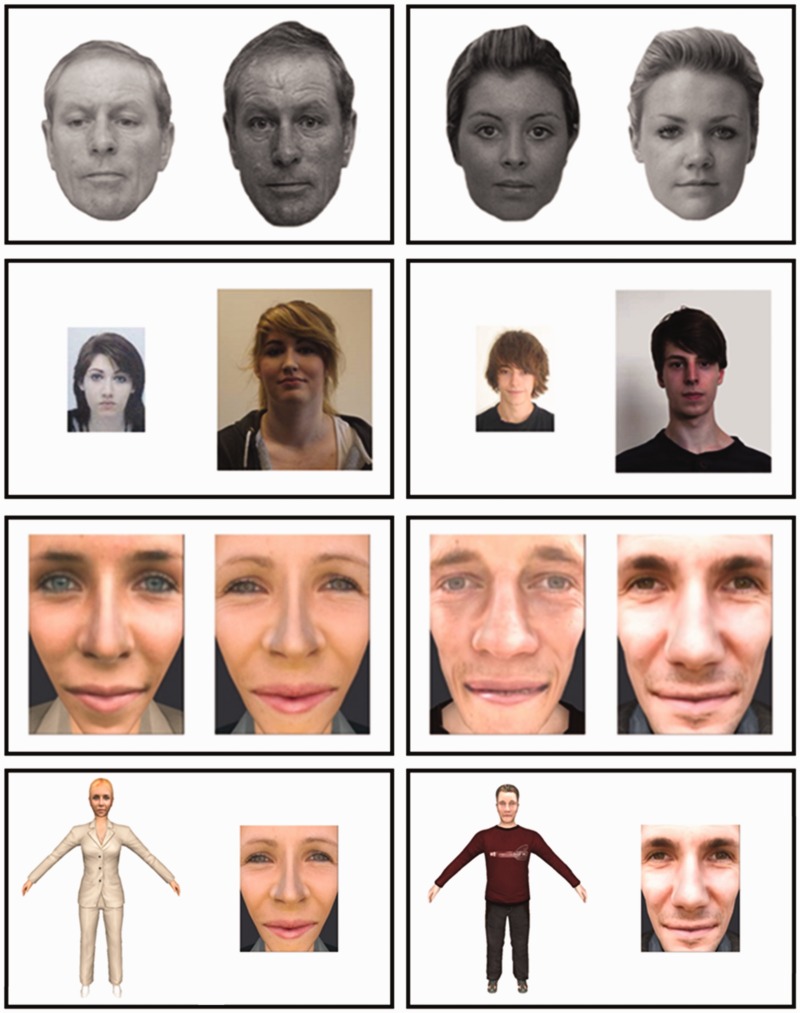
Example stimuli of match (left) and mismatch (right) trials for the GFMT (top row), KFMT (second row), avatar face portraits (third row), and whole avatar image to avatar face matching (bottom row).

The GFMT face pairs consist of images of faces taken from a frontal view displaying a neutral expression. Both images in a face pair are taken with different cameras and, in the case of identity matches, approximately 15 minutes apart. Each face image is cropped to show the head only and converted to greyscale with a resolution of 72 ppi. The dimensions of the faces range in width from 70 mm to 90 mm and in height from 85 mm to 125 mm and are spaced between 40 mm and 55 mm apart on screen. This study employed 20 identity match and 20 mismatch trials from the GFMT (for more information, see Burton et al., 2010). Example stimuli are shown in the top row of Figure 5.

##### The KFMT

Face pairs in the KFMT consist of an image from a student ID card, presented at a maximal size of 35 mm (w) × 47 mm (h), and a portrait photo, sized at 70 mm (w) × 82 mm (h) at a resolution of 72 ppi, spaced 75 mm apart. The student ID photos were taken at least 3 months prior to the face portraits and were not constrained by pose, facial expression, or image-capture device. The portrait photos depict the target’s head and shoulders from a frontal view while bearing a neutral facial expression and were captured with a high-quality digital camera. In this study, 20 identity match and 20 mismatch trials from the KFMT were employed (for more information, see Fysh & Bindemann, 2018). Example stimuli are shown in the second row of Figure 5.

##### Avatar face pairs

These stimuli are the same as those shown in Block 1 of Experiment 3 and consisted of 40 face pairs (20 identity matches, 20 mismatches), each depicting two avatar face portraits. For identity-match trials, the avatar faces in a pair were based on different source photographs, whereas two different identities were shown in identity-mismatch pairs. These faces were cropped to remove external features, such as hairstyle, and shown at a size of 70 mm (w) × 90 mm (h) and spaced 50 mm apart. Example stimuli are shown in the third row of Figure 5.

These three face-matching tasks (GFMT, KFMT, avatar pairs) were administered in separate blocks of 40 trials, which were presented in a counterbalanced order across participants. The procedure for all tasks was identical and presented using PsychoPy (Peirce, 2007). Thus, each trial begun with a 1-second fixation cross presented on a computer screen and was followed by a face pair, which participants were asked to classify as an identity match or mismatch as accurately as possible. Trial order was randomised within the blocks.

### Results

**Figure 6.**
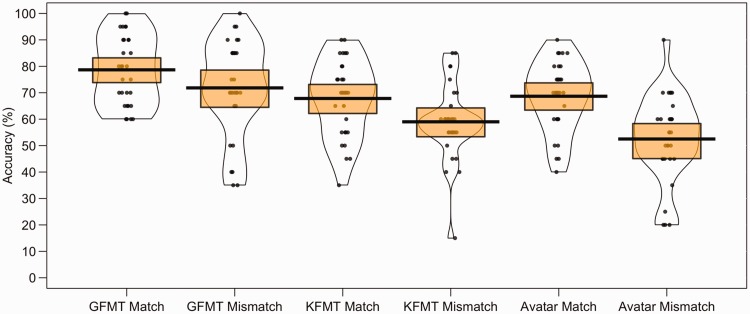
Percentage accuracy data for the GFMT, KFMT, and avatar face pairs in Experiment 4. The mean performance of each trial type is denoted by the black lines with the coloured boxes representing 95% confidence intervals. The black dots represent the accuracy of individual participants. The width of each violin represents the expected probability density of performance. GFMT = Glasgow Face Matching Test; KFMT = Kent Face Matching Test.

To compare performance across the three face-matching tasks, the mean percentage of correct match and mismatch responses was calculated for each participant. These data are illustrated in Figure 6. For match trials, the cross-subject mean accuracy was higher for the GFMT (*M* = 78.7%, *SD* = 13.2) than the KFMT (*M* = 67.8%, *SD* = 14.6) and the avatar face pairs (*M* = 68.7%, *SD* = 13.3). The same pattern was observed for mismatch trials, with higher accuracy for the GFMT (*M* = 71.8%, *SD* = 18.4) than the KFMT (*M* = 59.0%, *SD* = 14.4) and the avatar face pairs (*M* = 52.5%, *SD* = 16.6).

A 3 (task: GFMT, KFMT, avatar pairs) × 2 (trial type: match, mismatch) within-subjects ANOVA of these data confirmed a main effect of trial type, *F*(1, 29) = 8.83, *p* = .006, η_p_^2^ = .23, due to higher accuracy on match than mismatch trials. A main effect of task was also found, *F*(2, 58) = 34.70, *p* < .001, η_p_^2^ = .55. Paired-samples *t* tests (with alpha corrected to .017 [.05/3] for three comparisons) showed that accuracy was higher on the GFMT than both the KFMT, *t*(29) = 6.09, *p* < .001, *d* = 1.24, and the avatar pairs, *t*(29) = 7.87, *p* < .001, *d* = 1.57. There was no difference in accuracy between the KFMT and avatar pairs, *t*(29) = 1.58, *p* = .13, *d* = 0.32. The interaction of task and trial type was not significant, *F*(2, 58) = 2.35, *p* = .11, η_p_^2^ = .08.

A series of one-sample *t* tests was also conducted to determine whether accuracy was above chance (i.e., 50%) for the conditions. This was the case for match and mismatch trials on the KFMT, *t*(29) = 6.69, *p* < .001, *d* = 1.70 and *t*(29) = 3.42, *p* = .002, *d* = 0.87, and on the GFMT, *t*(29) = 11.90, *p* < .001, *d* = 3.03 and *t*(29) = 6.51, *p* < .001, *d* = 1.66. For avatar face pairs, accuracy was also above chance for match trials, *t*(29) = 7.68, *p* < .001, *d* = 1.96, but not for mismatch trials, *t*(29) = 0.83, *p* = .42, *d* = 0.21. A by-item inspection of these data shows a very broad range in accuracy for avatar mismatch face pairs, which suggests that mean chance performance masks items that are consistently classified correctly and also items that are classified consistently as incorrect. We return to further analysis of these data after Experiment 7, to demonstrate that these by-item differences for avatar stimuli are stable.

Overall, the mean percentage accuracy data show that accuracy on the GFMT is higher than for the KFMT and the avatar faces, which appear to be more evenly matched. While such general differences between these tasks were expected, the question of main interest in this experiment was whether performance on these tests is correlated. For match trials, Pearson’s correlations were obtained for the GFMT and KFMT, *r* = .580, *p* < .001, the GFMT and the avatar faces, *r* = .406, *p* = .03, and the KFMT and the avatar faces, *r* = .336, *p* = .05. Similarly, mismatch accuracy correlated for the GFMT and avatar faces, *r* = .550, *p* = .002, and the KFMT and the avatar faces, *r* = .407, *p* = .03. The correlation for mismatch trials on the GFMT and the KFMT did not reach significance, *r* = .333, *p* = .07.

### Discussion

This experiment correlated matching of avatar faces directly with two laboratory tests of face matching to determine whether identification of the avatars taps into the same processes as identification of real faces. Overall, accuracy was best with the highly optimised face pairs of the GFMT and comparable for the KFMT and the avatar faces. This finding makes good sense considering that the stimuli of the KFMT and those that were used to create the avatar face pairs captured identities across different days and more variable ambient conditions. Moreover, the similarity in performance across these tests suggest that low accuracy with the avatars reflects a difficulty in face matching that is comparable to the matching of challenging different-day face pairs (see Fysh & Bindemann, 2018; see also Megreya et al., 2013). Despite these differences in accuracy between the GFMT, KFMT, and the avatar faces, performance correlated well across the three tasks. This indicates that such avatar face pairs can provide a substitute to the matching of real faces for experimentation in VR.

## Experiment 5

The preceding experiments examine the matching of isolated face pairs. In contrast, identity matching in the VR environment requires comparison of a *person* with a face photograph. The inclusion of such body information reduces face size. This may affect identification, though it is unclear whether this would attenuate (see, e.g., Bindemann, Fysh, Sage, Douglas, & Tummon, 2017) or improve accuracy (see Bindemann et al., 2013). To explore this question under strictly controlled conditions, we conducted a further experiment in which the avatar matching stimuli comprised a whole person and a face photograph. As in Experiment 4, performance on this task was also compared with the GFMT and KFMT.

### Method

#### Participants

Thirty Caucasian participants from the University of Kent (11 men, 19 women), with a mean age of 21.0 years (*SD* = 2.9 years), participated for a small fee or course credit. None of these participants had participated in any of the preceding experiments.

#### Stimuli and Procedure

Stimuli and procedure were identical to Experiment 4, except for the following changes. The avatar matching stimuli comprised the same identities but now consisted of the image of a whole avatar (i.e., showing the entire body and the face) and an avatar face (for an illustration, see the bottom row of Figure 5). The whole avatar was sized to a height of 155 mm, with a body width of 35 mm (from hand to hand, 115 mm). This resulted in the face on the whole avatar to have dimensions of 20 mm (w) × 30 mm (h). By comparison, the isolated avatar face image in each stimulus pair measured 70 mm (w) × 90 mm (h) and was presented 30 mm apart from the whole avatar.

### Results

**Figure 7.**
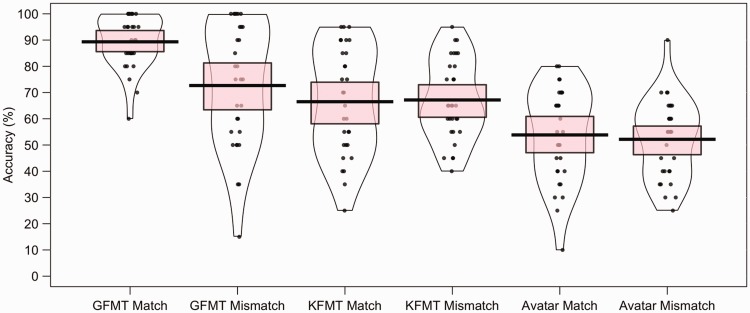
Percentage accuracy data for the GFMT, KFMT, and avatar stimulus pairs in Experiment 5. The mean performance of each trial type is denoted by the black lines with the coloured boxes representing 95% confidence intervals. The black dots represent the accuracy of individual participants. The width of each violin represents the expected probability density of performance. GFMT = Glasgow Face Matching Test; KFMT = Kent Face Matching Test.

The percentage accuracy data for this experiment are presented in Figure 7. For match trials, accuracy was higher for the GFMT (*M* = 89.3%, *SD* = 10.1) than the KFMT (*M* = 66.5%, *SD* = 20.5) and the avatar stimulus pairs (*M* = 53.8%, *SD* = 18.1). This pattern was also observed with identity mismatches, with highest accuracy for GFMT pairs (*M* = 72.7%, *SD* = 23.6), followed by the KFMT (*M* = 67.2%, *SD* = 15.4) and the avatar pairs (*M* = 52.2%, *SD* = 15.1).

A 3 (task: GFMT, KFMT, avatar) × 2 (trial type: match, mismatch) within-subjects ANOVA did not reveal a main effect of trial type, *F*(1, 29) = 1.47, *p* = .24, η_p_^2^ = .05, but showed a main effect of task, *F*(2, 58) = 75.27, *p* < .001, η_p_^2^ = .72, and an interaction, *F*(2, 58) = 9.32, *p* < .001, η_p_^2^ = .24. Simple main effects analysis was carried out to interpret this interaction. A simple main effect of trial type within the GFMT task was found, *F*(1, 29) = 9.53, *p* = .004, η_p_^2^ = .25, due to higher match than mismatch accuracy. There was no simple main effect of trial type within the KFMT, *F*(1, 29) = 0.01, *p* = .91, η_p_^2^ < .01, or avatar tasks, *F*(1, 29) = 0.10, *p* = .76, η_p_^2^ < .01.

In addition, a simple main effect of task within match trials was found, *F*(2, 28) = 98.89, *p* < .001, η_p_^2^ = .88. Paired-samples *t* tests (with alpha corrected to .017 [.05/3] for three comparisons) showed accuracy on the GFMT was higher than for both the KFMT and the avatar task on match trials, *t*(29) = 7.51, *p* < .001, *d* = 1.39 and *t*(29) = 13.39, *p* < .001, *d* = 2.39, respectively. The KFMT was also performed more accurately than the avatar task on match trials, *t*(29) = 3.49, *p* = .002, *d* = 0.65.

Similarly, a simple main effect of task within mismatch trials was also found, *F*(2, 28) = 32.84, *p* < .001, η_p_^2^ = .70. Paired-samples *t* tests (with alpha corrected to .017 [.05/3] for three comparisons) showed accuracy was higher on the GFMT and KFMT than the avatar task for this trial type, *t*(29) = 6.48, *p* < .001, *d* = 1.02 and *t*(29) = 5.99, *p* < .001, *d* = 0.97, respectively. There was no difference in mismatch trial accuracy between the GFMT and KFMT, *t*(29) = 1.47, *p* = .15, *d* = 0.27.

Finally, a series of one-sample *t* tests was also conducted to determine whether accuracy was above chance (i.e., 50%) for the conditions. This was the case for match and mismatch trials on the GFMT, *t*(29) = 21.41, *p* < .001, *d* = 5.46 and *t*(29) = 5.26, *p* < .001, *d* = 1.34, and the KFMT, *t*(29) = 4.41, *p* < .001, *d* = 1.12 and *t*(29) = 6.13, *p* < .001, *d* = 1.56. In contrast, accuracy for the avatar pairs did not exceed chance for match trials, *t*(29) = 1.16, *p* = .26, *d* = 0.30, nor mismatch trials, *t*(29) = 0.79, *p* = .43, *d* = 0.20. However, a by-item inspection of these data again shows a very broad range in accuracy, suggesting that mean performance masks consistent correct and incorrect classifications of avatar items (further analysis provided after Experiment 7). Moreover, Pearson correlations revealed that match accuracy correlated across all combinations of the GFMT, KFMT, and the avatar stimuli, all *r*s ≥ .474, all *p*s ≤ .008, as did accuracy for mismatch trials, all *r*s ≥ .514, all *p*s ≤ .004.

### Discussion

This experiment replicates the main findings of Experiment 4, by revealing that performance for matching GFMT, KFMT, and avatar faces correlates consistently. This provides further evidence that identification across these tasks is based on similar processes. However, in contrast to Experiment 4, which displayed only avatar faces, matching avatar faces to whole persons was more difficult in Experiment 5, and accuracy was low. We attribute this poor performance to the size of the whole body stimuli, which resulted in a compression of the facial information (see bottom row of Figure 5). This raises the question of whether these avatars provide sufficient information for person identification during immersion in a VR airport environment. This was examined in the final phase of this study.

## Phase 3: Face Matching in VR

**Figure 8.**
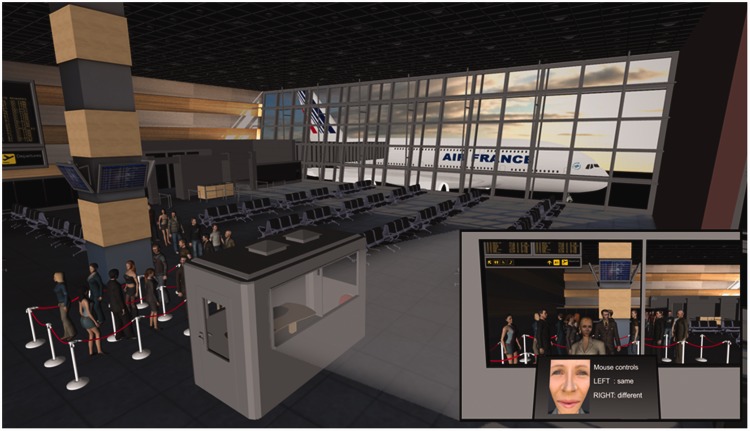
An overhead view of the virtual reality airport. Inset (bottom right) displays the viewpoint of the participants from the passport control booth, when processing the queue in Experiment 6.

In the final phase, we examined avatar identification in VR, by constructing a passport control desk in an airport arrivals hall. This environment comprised an airport lounge, with seating and rope queue barriers to channel travellers to a passport control booth. Visual cues were incorporated to convey clearly to participants that this is an airport environment, such as departure boards and a waiting aeroplane within view of the passport control desk area. This environment is illustrated in Figure 8.

Participants were immersed in this environment and asked to take on the role of passport officers in the control booth, by processing a queue of travellers by identity matching a face photograph to an avatar’s appearance (see inset of Figure 8). Animated avatars queued in line and then approached the booth individually to be processed. After participants made an identification decision, the avatar would then walk away, with stimuli classified as identity matches proceeding past the booth and towards an exit at the back of the airport hall, while stimuli classified as mismatches would walk into a waiting area to the side of the control point.

## Experiment 6

In Experiment 6, we employed this airport environment to investigate face matching in VR. We employed the same avatar identities as in the preceding experiments and specifically sought to examine the accuracy levels that participants achieve in this task.

### Method

#### Participants

Thirty Caucasian participants from the University of Kent (7 men, 23 women), with a mean age of 21.6 years (*SD* = 4.1 years), took part for a small fee or course credit. None of these participants had participated in the preceding experiments. Owing to the use of VR equipment, no persons with epilepsy or who were liable to motion sickness were recruited. Before immersion in VR, participants were briefed about potential side effects of using VR, such as discomfort from wearing the headset and symptoms of motion sickness, and health and safety procedures.

#### Stimuli and Procedure

The stimuli consisted of the same avatar-face pairings that were employed in Experiment 5, comprising 20 matches and 20 mismatches. These were displayed in the VR environment using Vizard 5 and an Oculus Rift DK2 headset, with a resolution of 960 × 1,080 pixels per eye with 100° field of view and an image refresh rate of 75 Hz.

On immersion in the VR environment, participants found themselves seated in the passport control booth, which was equipped with a desk and desktop PC. A group of 40 avatars then arrived in the airport hall and queued at the control desk, with one avatar at a time approaching the participants. As each avatar approached, their *passport photograph* would appear on the screen of the desktop PC. Participants were asked to compare this image with the face of the presenting avatar, and make identity-match or mismatch decisions via button presses on a computer mouse. Once a response was registered, the avatar would move past the control desk to exit the airport hall (if classified as a match) or would depart to the side of the airport hall into a waiting area (if classified as a mismatch). At this point, the next avatar would approach the control desk, prompting the start of the next trial. Presentation of avatars was randomised. Accuracy of response was emphasised, and there was no time restriction for task completion.

### Results

**Figure 9.**
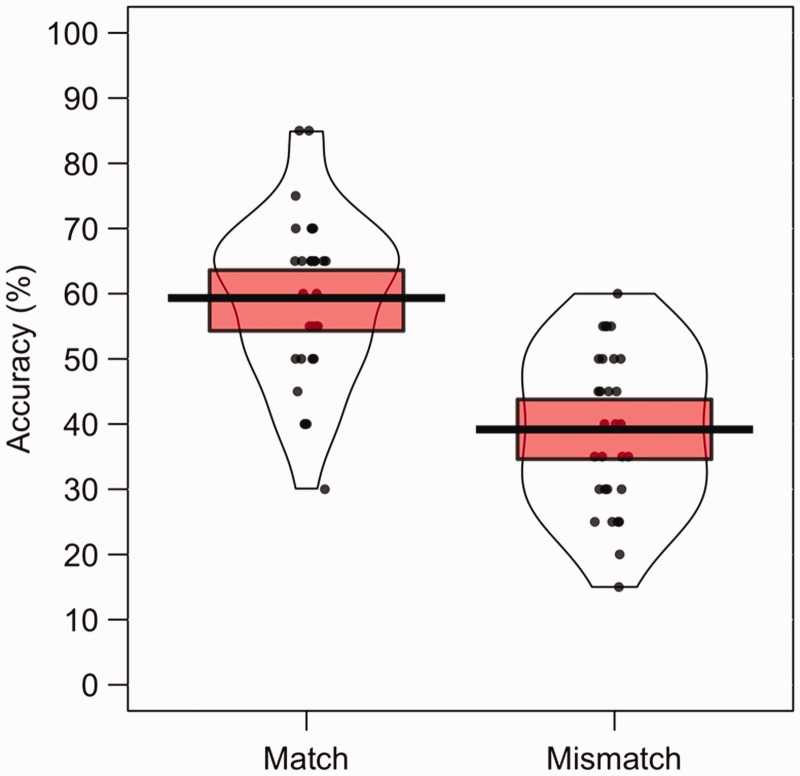
Percentage accuracy data for Experiment 6. The mean performance of each trial type is denoted by the black lines with the coloured boxes representing 95% confidence intervals. The black dots represent the accuracy of individual participants. The width of each violin represents the expected probability density of performance.

The percentage accuracy data for this VR experiment are illustrated in Figure 9. A paired-sample *t* test showed that accuracy was higher on match trials (*M* = 59.3%, *SD* = 13.0) than mismatch trials (*M* = 39.2%, *SD* = 12.0), *t*(29) = 5.29, *p* < .001, *d* = 1.59. In addition, one-sample *t* tests showed that performance was above chance (50%) on match trials, *t*(29) = 3.94, *p* < .001, *d* = 1.00, but below chance on mismatch trials, *t*(29) = 4.93, *p* < .001, *d* = 1.26. However, by-item inspection of these data again shows a very broad range in accuracy for mismatch stimuli (further analysis provided after Experiment 7).

Cross-experiment analyses were conducted to examine how performance for this face-to-avatar matching in VR compared with the still image avatar matching of Experiment 4 (face-to-face matching: match accuracy *M* = 68.7%, *SD* = 13.3; mismatch accuracy *M* = 52.5%, *SD* = 16.6) and Experiment 5 (face-to-body matching: match accuracy *M* = 53.8%, *SD* = 18.1; mismatch accuracy *M* = 52.2%, *SD* = 15.1). A 3 (stimulus type: face-to-face, face-to-body, face-to-avatar) × 2 (trial type: match, mismatch) mixed-factor ANOVA showed main effects of trial type, *F*(1, 87) = 22.73, *p* < .001, η_p_^2^ = .21, and stimulus type, *F*(2, 87) = 16.25, *p* < .001, η_p_^2^ = .27, and an interaction between these factors, *F*(2, 87) = 4.47, *p* = .01, η_p_^2^ = .09.

To interpret this interaction, simple main effects analyses were carried out. A simple main effect of trial type was found for face-to-face matching (Experiment 4), *F*(1, 87) = 12.34, *p* < .001, η_p_^2^ = .12, and face-to-avatar matching (Experiment 6), *F*(1, 87) = 19.20, *p* < .001, η_p_^2^ = .18, both due to higher match than mismatch accuracy. There was no simple main effect of trial type for face-to-body matching (Experiment 5), *F*(1, 87) = 0.13, *p* = .72, η_p_^2^ < .01.

In addition, a simple main effect of stimulus type within match trials was found, *F*(2, 87) = 7.52, *p* < .001, η_p_^2^ = .15. Paired-samples *t* tests (with alpha corrected to .017 [.05/3] for three comparisons) showed that face-to-face matching was performed more accurately than both face-to-body matching, *t*(58) = 3.62, *p* < .001, *d* = 0.92, and face-to-avatar matching, *t*(58) = 2.75, *p* = .008, *d* = 0.70. There was no difference in accuracy between these latter two stimulus types on match trials, *t*(58) = 1.35, *p* = .18, *d* = 0.34.

A simple main effect of stimulus type within mismatch trials was also found, *F*(2, 87) = 8.02, *p* < .001, η_p_^2^ = .16. Paired-samples *t* tests (with alpha corrected to .017 [.05/3] for three comparisons) showed accuracy was higher for both face-to-face and face-to-body matching over face-to-avatar matching, *t*(58) = 3.56, *p* < .001, *d* = 0.91 and *t*(58) = 3.68, *p* < .001, *d* = 0.94, respectively. No difference in accuracy was found between face-to-face and face-to-body matching on mismatch trials, *t*(58) = 0.08, *p* = .94, *d* = 0.02.

### Discussion

The results from this experiment indicate an increase in task difficulty when face matching is performed in VR. The accuracy of avatar matching, particularly on mismatch trials, was considerably lower in the VR environment than when the same stimuli were presented in 2D and in isolation in Experiments 4 and 5. Considering this low accuracy, we modified our paradigm for a final experiment in an attempt to improve performance.

## Experiment 7

**Figure 10.**
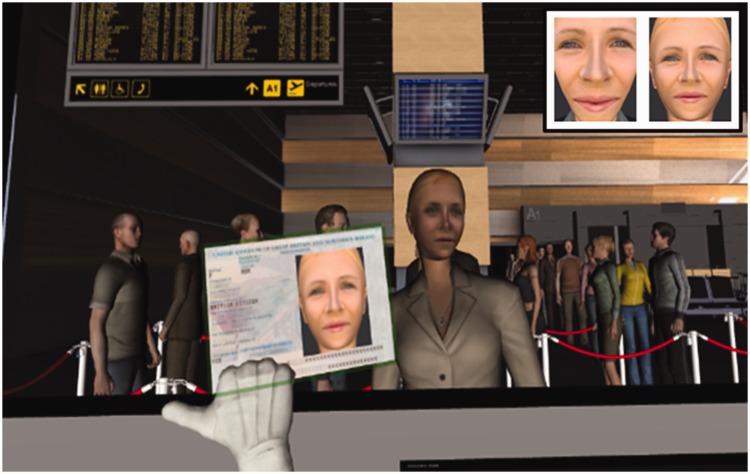
Improved interactivity of airport environment in Experiment 7. Inset (top right) displays an avatar face portrait from Experiment 6 (left) alongside its updated image for Experiment 7 (right).

In this experiment, we attempted to optimise the VR paradigm to improve face-matching performance. We replaced the Oculus Rift DK2 headset with an HTC Vive, which provides greater screen resolution (960 × 1,080 pixels per eye vs. 1,080 × 1,200 pixels per eye). The HTC Vive is also equipped with handheld controllers to enable participants to interact better with the environment. We utilised the controllers to allow participants to hold the passports of travellers in the VR environment. This enabled participants to bring these closer to their own face, thus increasing the size and resolution of these images for comparison, as well as to hold the passport photos next to the travellers to facilitate face matching (see Figure 10). As a final change, we rerecorded the face image for the photo identities in VR. The software models convexity by elongating face shape as viewing distance decreases. As a result of this, the avatar face stimuli were narrow in appearance in the preceding experiments, particularly near the chin region. We rerecorded these images from greater distance to produce a more natural, rounded appearance (see inset of Figure 10). We then examined whether face-matching performance in the VR environment was improved as a result of these changes.

### Method

#### Participants

Thirty Caucasian participants from the University of Kent (7 men, 23 women) with a mean age of 20.3 years (*SD* = 2.8 years) participated for a small fee or course credit. None of these participants had participated in the preceding experiments. No persons with epilepsy or who were liable to motion sickness were recruited. All participants were given a health and safety briefing prior to immersion in the VR.

#### Stimuli and Procedure

The stimuli consisted of the same avatar identities as in Experiment 6, but the images for the passport photographs were rerecorded at a great viewing distance to produce faces with a more natural, rounded face shape (see inset of Figure 10). The size of these images was maintained at 438 (w) × 563 (h) pixels at a resolution of 150 ppi. The procedure was identical to Experiment 6 except that the Oculus Rift DK2 headset was replaced with an HTC Vive, which has an improved resolution of 1,080 × 1,200 pixels per eye with 110° field of view with a faster image refresh rate of 90 Hz. In addition, two handheld controllers were utilised as controls for this experiment.

On each trial, the passport face image was no longer presented on the desktop PC in the control booth but was inserted into a passport-style card, which could be picked up by participants using a handheld controller. This enabled participants to hold the passport images closer to their own eyes or next to the avatar’s head to facilitate identity comparison. The handheld controllers were also employed to record participants’ responses, with button presses on the right-hand controller indicating identity matches and on the left-hand controller indicating mismatches.

### Results

**Figure 11.**
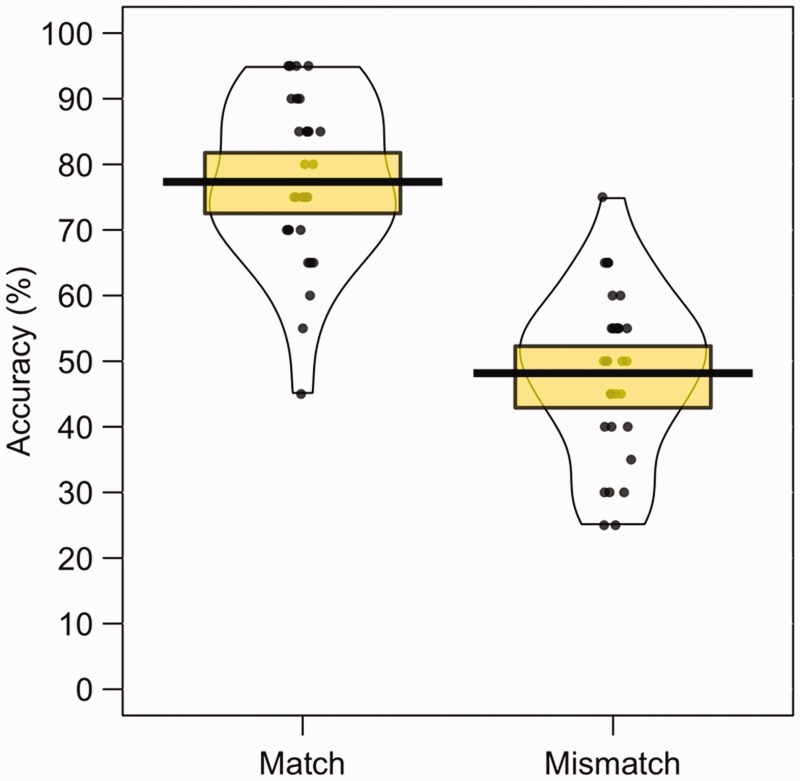
Percentage accuracy data for Experiment 7. The mean performance of each trial type is denoted by the black lines with the coloured boxes representing 95% confidence intervals. The black dots represent the accuracy of individual participants. The width of each violin represents the expected probability density of performance.

As in all preceding experiments, accuracy was higher for match trials (*M* = 77.3%, *SD* = 12.6) than mismatch trials (*M* = 48.2%, *SD* = 12.6), *t*(29) = 7.28, *p* < .001, *d* = 2.28, as illustrated in Figure 11. In addition, match accuracy was reliably above chance level (i.e., 50%), *t*(29) = 11.90, *p* < .001, *d* = 3.03, whereas mismatch accuracy was not, *t*(29) = 0.80, *p* = .43, *d* = 0.20. Again, however, by-item inspection of the mismatch data shows broad differences between items (further analysis provided after this experiment).

To determine whether the adjustments to the VR paradigm successfully reduced the difficulty of the task, a 2 (environment: Experiment 6, Experiment 7) × 2 (trial type: match, mismatch) mixed-factor ANOVA was conducted. This showed a main effect of trial type, *F*(1, 58) = 79.67, *p* < .001, η_p_^2^ = .58, due to higher accuracy on match trials than mismatch trials. A main effect of environment was also found, *F*(1, 58) = 63.27, *p* < .001, η_p_^2^ = .52, reflecting higher accuracy in Experiment 7. The interaction between trial type and experiment was not significant, *F*(1, 58) = 2.65, *p* = .11, η_p_^2^ = .04.

### Discussion

This experiment demonstrates that the improvements to the VR paradigm enhanced accuracy. This improvement was particularly marked on match trials, where accuracy reached 77%. Mismatch performance was enhanced too but remained particularly difficult in the VR paradigm, at 48% accuracy. This is a limiting factor for research on unfamiliar face matching, considering the important role that these trials hold for person identification at passport control in the real world (see, e.g., Fysh & Bindemann, 2017a). However, previous research on face matching demonstrates that considerable variation in accuracy can exist across items, to the point where some items may be consistently classified incorrectly (see Fysh & Bindemann, 2018). In turn, this raises the possibility that even though mean performance on mismatch trials does not exceed 50%, a substantial proportion of these may nonetheless be classified with high accuracy. A cursory analysis of such by-item differences was provided in Experiments 4 to 7, which revealed broad differences in accuracy between individual items. To explore whether these by-item differences are stable, we performed correlational comparisons across Experiments 4 to 7.

## Comparison of Items Across Experiments

**Figure 12.**
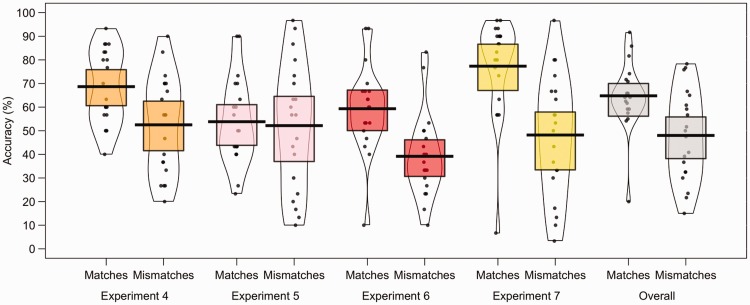
Percentage accuracy data by avatar item for Experiments 4 to 7. The mean performance of each avatar trial type is denoted by the black lines with the coloured boxes representing 95% confidence intervals. The black dots represent accuracy for individual face pairs. The width of each violin represents the expected probability density of performance.

To analyse accuracy for individual items, the mean accuracy for each stimulus pair was compared across experiments (i.e., for face-to-face pairs in Experiment 4, face-to-body in Experiment 5, and face-to-avatar in Experiments 6 and 7). These scores are illustrated in Figure 12 and reveal considerable variation in accuracy across items. In Experiment 4, for example, this variation is such that accuracy for individual match items ranges from 40% to 93% and from 20% to 90% for mismatch items. These differences were even more marked by Experiment 7, in which by-item accuracy ranged from 7% to 97% for match stimuli and from 3% to 97% for mismatch stimuli. This range in accuracy indicates that some items were consistently classified correctly, whereas other yielded consistently incorrect decisions. A reliability analysis was conducted across Experiments 4 to 7, with Cronbach’s alpha showing accuracy for match items, α = .66, to be more consistent than accuracy for mismatch items, α = .55. However, despite the variation in item accuracy, strong positive correlations were obtained for by-item accuracy across Experiments 4 to 7 (see Table 1).

**Table 1.** Mean Accuracy and Correlations Between Experiments Across All Avatar Items.

Trial type	Experiment	*M*	*SD*	Correlation coefficients (*r*)			
				4	5	6	7
Overall	4	60.7	19.9	–			
	5	53.0	22.9	.552***	–		
	6	49.2	20.7	.539***	.484**	–	
	7	62.8	27.7	.627***	.553***	.647***	–
Match	4	68.7	15.8	–			
	5	53.8	18.4	.499*	–		
	6	59.3	18.5	.255	.515*	–	
	7	77.4	21.2	.394	.423	.741***	–
Mismatch	4	52.6	20.7	–			
	5	52.2	27.1	.639**	–		
	6	39.1	17.9	.566**	.571**	–	
	7	48.2	25.9	.613**	.752***	.342	–

*p < .05. **p < .01. ***p < .001.

For match items, by-item accuracy correlated well for each progression towards face matching in VR. Accuracy when matching two avatar face portraits (Experiment 4) positively correlated with the accuracy of matching one of these avatar face images with an avatar body image (Experiment 5), *r* = .499, *p* = .03. When this avatar face-body matching was conducted in VR (Experiment 6), accuracy correlated with its still image counterpart (Experiment 5), *r* = .515, *p* = .02. Item accuracy in the original VR paradigm (Experiment 6) also correlated strongly with item accuracy when the VR paradigm was improved in Experiment 7, *r* = .741, *p* < .001. However, all other correlations between experiments were nonsignificant, all *r*s ≤ .423, all *p*s ≥ .06.

Accuracy for many mismatch items was lower than for any of the match items across all experiments but correlated strongly across all comparisons between Experiments 4 to 7, all *r*s ≥ .566, all *p*s < .009, except between the two VR experiments (Experiments 6 and 7), *r* = .342, *p* = .14. We attribute this discrepancy to the improvement gains possible from Experiment 6 to Experiment 7, which was much greater for some items compared with others.

Overall, the finding that accuracy for items is highly consistent across experiments under the conditions investigated here provides a potential solution to the poor mean accuracy in the mismatch condition. To model the real world of passport control, match trials should occur with much greater frequency than mismatch trials in experiments on unfamiliar face matching (see, e.g., Bindemann, Avetisyan, & Blackwell, 2010; Fysh & Bindemann, 2017b, 2018; Papesh & Goldinger, 2014; Susa, Michael, Dessenberger, & Meissner, 2018). One way to address the poor mean accuracy across mismatch items in VR here could therefore be to select the mismatches with the highest by-item accuracy for further experimentation. Ultimately, however, we think that this problem will be addressed also through future development of higher quality avatars, which will enhance accuracy of avatar facial identification.

## General Discussion

This study explored the feasibility of conducting face-matching experiments in VR. This exploratory study is the first of its kind in this field and was conducted in three phases. The first phase investigated whether avatar faces can provide suitable replacements for face photographs, by asking participants to perform avatar-to-photograph identity matching. Accuracy was high when stimuli displayed avatar faces alongside the photograph from which these were derived (Experiment 1). This image-specific identity matching indicates that the avatars successfully captured their source face photograph. Matching accuracy also exceeded chance on mismatch trials, in which two different identities were shown (Experiments 1 and 2), and with different-image identity matches, in which an avatar face was shown alongside a different source photograph of the same identity (Experiment 2). This indicates that the avatars captured not only the source image but also the identity of these targets. The final validation experiment in this first phase investigated whether accuracy when matching avatar-to-avatar would be consistent with the matching of pairs of photographs (Experiment 3). Despite avatar matching being a more difficult task than photograph matching, participant accuracy exceeded chance and correlated for the two image types. The experiments in this phase therefore demonstrate that our avatar stimuli can provide a suitable substrate to study such face identification processes in VR.

The second phase sought to validate the avatar stimuli further by correlating performance in avatar-to-avatar matching with two established tests of face-to-face matching (the GFMT, see Burton et al., 2010; and the KFMT, see Fysh & Bindemann, 2018). Avatar matching correlated consistently with these face tests, both when pairs of avatar faces were shown (Experiment 4) and when an avatar face was paired with a whole avatar body (Experiment 5). This indicates that matching of avatars and of real face photographs reflect similar cognitive processes.

In the final phase, we examined avatar identification with a VR airport environment, in which participants took up the role of passport officer at a control point. A first run of this paradigm proved difficult, with average accuracy for identity-mismatch trials below chance level (Experiment 6). The application of higher resolution VR equipment, and modifications to the experimental paradigm that allowed participants to view avatar faces more flexibly, improved accuracy (Experiment 7). However, accuracy on mismatch trials remained near chance. We therefore performed a by-item analysis to determine whether individual mismatch trials were classified consistently. This analysis revealed strong correlations across Experiments 4 to 7, indicating that by-item classification was robust across experiments. This by-item data also revealed that some mismatch trials were classified consistently with low but some also with high accuracy. Considering that mismatches should occur with much lower frequency than match trials when one seeks to mimic real-world conditions (see, e.g., Bindemann, Avetisyan, & Blackwell, 2010; Fysh & Bindemann, 2017b, 2018; Papesh & Goldinger, 2014; Susa et al., 2018), the by-item data could therefore provide a basis for selecting mismatch stimuli that give rise to high (or low) accuracy for further experimentation.

Overall, these data provide proof of principle for the use of VR for face-matching research. While the generation of VR explored here does not yet meet real-world detail, realism, and identification accuracy, the rapid development of this technology provides a promising outlook for future research. This opens up many avenues for face-matching research, by facilitating the study of new environment and social interaction factors that may be relevant in real-world operational settings. With regard to passport control, for example, it is possible that nonfacial cues, such as body language, draw attention to potential impostors and could also support identification decisions (Rice, Phillips, Natu, et al., 2013; Rice, Phillips, et al., 2013). Similarly, environmental factors, such as the mere presence of passenger queues, might impair identification by exerting time pressure on passport officers (see, e.g., Bindemann et al., 2016; Fysh & Bindemann, 2017b; Wirth & Carbon, 2017). Crowd dynamics, such as animated body language throughout queues might also signal impatience to passport officers and exert further pressure. Crucially, such factors cannot be captured well by current laboratory paradigms and are practically impossible to study in real life owing to the importance of person identification at passport control. The current study demonstrates the feasibility of VR for studying and understanding such phenomena, which can only improve as the technology continues to develop.

We note that our study still represents a relatively simple approach for the implementation of such experiments. For example, we created our avatar faces by a rather simplistic process that was based on the superimposition of 2D photographs on existing avatar structures. In future, we anticipate that the 3D scanning of faces and the rigging of this information into avatars as well as further development of VR technology will result in person stimuli and environments that provide increasingly closer representations of reality. This should support experimentation by further enhancing identification of identity matches and mismatches. Ultimately, we expect VR to become an important research tool for investigating face perception in complex and realistic environments, with increasing collaboration between researchers and developers accelerating advancement in this field.

## Supplemental Material

Supplemental material for Facial Identification at a Virtual Reality Airport Click here for additional data file. Supplemental Material for Facial Identification at a Virtual Reality Airport by Hannah M. Tummon, John Allen and Markus Bindemann in i-Perception
